# Planting grass enhances relations between soil microbes and enzyme activities and restores soil functions in a degraded grassland

**DOI:** 10.3389/fmicb.2024.1290849

**Published:** 2024-02-15

**Authors:** Minghui Zhang, Zhuo Li, Bin Zhang, Ruohui Zhang, Fu Xing

**Affiliations:** ^1^Key Laboratory of Vegetation Ecology, Ministry of Education, Institute of Grassland Science, Northeast Normal University, Changchun, China; ^2^Jilin Songnen Grassland Ecosystem National Observation and Research Station, Changchun, China

**Keywords:** soil microbial functional groups, extracellular enzyme activities, grassland degradation, soil functions, *Leymus chinensis*

## Abstract

**Introduction:**

Forage culture is a common way to restore degraded grasslands and soil functions, in which the reconstruction of the soil microbial community and its relationship with extracellular enzyme activity (EEAs) can characterize the recovery effects of degraded grasslands. However, the impacts of forage culture on the interaction between soil microbes and EEAs and whether the recovery effect of soil functions depends on the varying degradation statuses remain unclear.

**Methods:**

We conducted a plantation of a dominant grass, *Leymus chinensis*, in the soil collected from severe, moderate, light, and non-degradation statuses in the Songnen grassland in northeastern China. We measured soil microbial diversity and soil EEAs, and predicted microbial functional groups using FUNGuild.

**Results:**

The results showed that *L. chinensis* culture promoted soil bacterial alpha diversity and soil EEAs only in the moderate degradation status, indicating a dramatic dependence of the recovery effects of the grass culture on degradation status of the grassland. After planting *L. chinensis* for 10 weeks, a decreasing trend in the chemoheterotrophy and nitrate-reduction microbial functional groups was found. In contrast, the abundance of the nitrogen (N)-fixing microbial functional group tended to increase. The positive correlation between soil EEAs and the nitrate-reduction and N-fixing microbial functional groups was enhanced by planting *L. chinensis*, indicating that grass culture could promote soil N cycle functions.

**Conclusion:**

We illuminate that grass culture may promote the restoration of soil functions, especially soil N cycling in degraded grasslands, and the recovery effect may depend on the grassland degradation status. We emphasized that selection of the plant species for restoration of grasslands needs to consider the restoration effects of microbial functional groups and soil functions.

## Introduction

Grassland ecological restoration refers to the recovery process of degraded grassland to the non-degradation statuses, including plant community restoration and soil function recovery (Kardol and Wardle, 2010; Jing et al., 2015; Liu et al., 2019). Plant community restoration focuses on increasing plant community productivity and local native vegetation reestablishment (Jing et al., 2015). Soil function recovery emphasizes soil microbial community restructure and enhancing soil extracellular enzyme activities (EEAs) (Kardol and Wardle, 2010). Previous studies showed that reseeding forage is one of the effective ways to restore degraded grasslands (Fry et al., 2017; Liu et al., 2019). Forage culture effectively increased the vegetation biomass and richness and thus accelerated the plant community restoration in degraded grassland (Wang et al., 2017). Moreover, soil microbial communities are usually associated with plant diversity and biomass, and changes in plant communities during grassland restoration can affect the structure of microbial communities (Li et al., 2016, 2022; Xun et al., 2018). Therefore, the increased plant total biomass caused by forage culture may regulate soil microbial communities and EEAs. This regulation effect may benefit soil function recovery in degraded grassland, whereas the importance of forage culture on soil microbial community and EEAs has only recently been recognized.

Soil microbial community restructure is fundamental for the ecological recovery of degraded grassland soil, and the microbial community structure is influenced by the degree of grassland degradation (Chodak and Niklińska, 2010; Li et al., 2016). Generally, different degradation statuses significantly change plant biomass and composition (Liu et al., 2021). These plants regulate microorganisms by litter and root exudates, thus forming different microbial community in soils at different degradation statuses (Li et al., 2016). Furthermore, grassland degradation are accompanied by soil nutrient loss and soil salinization (Akiyama and Kawamura, 2007; Singh K, 2015). Low soil nutrient concentrations and high soil salinization in severe degradation grasslands can decrease microbial abundance and diversity (Singh JS, 2015; Che et al., 2019). As a result, grassland degradation significantly changed soil microbial characteristics, and forage culture may have different regulatory effects on microbial community in the different degraded grasslands. For instance, reseeding *Rhodiola kirilowii* promotes soil microbial biomass and activities in the degraded desertified alpine meadow (Liu et al., 2019); nevertheless, planting *Corispermum macrocarpum* did not significantly affect bacterial abundance and Shannon index in the severely degraded sandy grasslands (Wang et al., 2017). Therefore, forage culture can only reconstruct microbial communities in some suitable degradation statuses. Choosing suitable degradation statuses for reseeding forage is the key to improving the efficiency of microbial community restructure in degraded grassland.

Soil microbial functional groups refer to a collection of microorganisms with specific biological functions (Li et al., 2023). These microbial functional groups are essential in regulating soil functions (Wang et al., 2021). As different plants have different physiological characteristics, changes in plant biomass and species are likely to alter the activity and abundance of microbial functional groups (Wang et al., 2021). Therefore, forage culture may influence the composition and abundance of microbial functional groups, and this regulatory effect is crucial for the recovery of soil functions in degraded grassland. For example, pioneer plants (*Serianthes calycina* and *Gymnostoma webbianum*) regulated bacterial functional groups involved in the N-cycle in the Nickel mine soils (Héry et al., 2005). Moreover, the effects of forage culture on microbial functional groups may drive the progress of grassland ecological restoration (Fry et al., 2017; Li et al., 2021). A study showed that planting gramineous forage (*Elymus nutans*) increased the diversity and activity of asymbiotic nitrogen-fixing bacteria, which may promote soil function recovery and degraded grassland restoration (Li et al., 2021). However, forage culture rapidly increases plant biomass, which may cause an increase in plant pathogen abundance, thus inhibiting plant growth and grassland restoration rates (Kardol and Wardle, 2010). Accordingly, the effects of forage culture on microbial functional groups in degraded grassland remain to be clarified. For instance, it is unclear whether different degradation status affects the efficiency of forage culture in regulating microbial functional groups. Such information is essential for improving and enhancing the recovery efforts of soil functions in degraded grassland.

Soil EEAs can reflect the activity of microbial-mediated soil functions (Chodak and Niklińska, 2010; Liu et al., 2021). In grassland restoration, plants regulate soil microbial functional groups by releasing root exudates and degrading litter (Steinauer et al., 2016; Li et al., 2021). Subsequently, microbial functional groups produce soil extracellular enzymes, and the activity of these enzymes is positively correlated with soil functions (Liu et al., 2021). For example, soil microbes regulate the activity of β-1,4-glucosidase and β-1,4-N-acetyl-glucosaminidase enzymes, which may influence soil carbon (C) and nitrogen (N) cycling functions (Song et al., 2019; Chuan et al., 2020). Therefore, the interaction between microbial functional groups and EEAs is a primary indicator of the recovery of soil functions in degraded grasslands (Burns et al., 2013; Liu et al., 2021). Furthermore, both microbial functional groups and soil EEAs are affected by the soil environment (Allison et al., 2010; Liu et al., 2021). The different soil environments in the varying grassland degradation statuses may interfere with the interaction between soil microbial communities and EEAs (Allison et al., 2010; Burns et al., 2013). For instance, high soil pH in the degraded grassland could inhibit the activity of microbial functional groups and soil EEAs (Liu et al., 2019; Wang et al., 2021). Moreover, forage culture may promote soil EEAs in degraded grasslands (Parraga-Aguado et al., 2013). For example, forage culture significantly enhanced plant biomass in degraded grasslands (Li et al., 2022). The higher plant biomass produces more litter and root exudates, which promotes soil EEAs (Steinauer et al., 2016; Liu et al., 2021). These effects can promote soil functions recovery and provide beneficial environments for subsequent plant growth (Burns et al., 2013; Zhang et al., 2015; Song et al., 2019; Li et al., 2022). However, the restoration effects of forage culture may vary significantly due to the differences in soil physicochemical characteristics and soil microbial functional groups at different succession stages of grassland soils (Singh K, 2015). For instance, ryegrass plantation (*Lolium perenne*) promotes the activity of soil acid phosphatase enzyme by producing root exudations, and the regulation effects of ryegrass plantation on soil EEAs varies among different soil types (Chen et al., 2004). Accordingly, the degree of grassland degradation may interfere with the regulation effects of forage culture on soil microbes and EEAs. Nevertheless, few studies have examined how forage culture affects the interaction between microbial communities and soil EEAs in the varying grassland degradation statuses.

*Leymus chinensis* is a perennial rhizomatous bunchgrass widely distributed and dominated in the Songnen grassland in northeastern China (Liu et al., 2021; Zhang et al., 2022). *L. chinensis* has a strong tolerance to drought, high soil pH, and low soil fertility (Wang and Ripley, 1997; Wang et al., 2004). These characteristics result in *L. chinensis* being a dominant species in the Songnen grassland and a pioneer species in the local grassland restoration. Therefore, *L. chinensis* culture is a practical approach to restoring the degraded grasslands in this area (Wang et al., 2004). This study aims to detect how *L. chinensis* planting regulates the interaction between soil microbes and extracellular enzymes in the varying degradation statuses (i.e., severe, moderate, light, and non-degradation status). We conducted the *L. chinensis* culture experiment using soils from varying degradation statuses and measured the soil microbial characteristics and EEAs. We hypothesized that (1) the regulation effects of *L. chinensis* planting on soil microbial communities, including plant probiotics and pathogens and soil EEAs, depend on degraded status, and (2) *L. chinensis* planting enhances the correlation between soil microbial communities and EEAs and thus promoting soil N cycle function in the degraded grassland.

## Materials and methods

### Sample area description

Our field survey and soil collection are concluded at the Tongyu Semi-Arid Climate-Environment Field Station (122°52′E, 44°25′N), part of the Chinese Academy of Sciences. The Tongyu station covers an area of 47.7 hm^2^, which is located on the Songnen Plain, northeastern China. This area is described as a meadow steppe, and the dominant species is *L. chinensis*. The mean annual precipitation is 404 mm, with 80% falling during the growing season (from May to September). The mean annual temperature is 5.7°C. The soil types in this area are sandy soils, slight chernozem soils, salty alkaline soils, and meadow soils (Gao et al., 2019; Liu et al., 2021).

Since 2006, the meadow steppe within Tongyu station has been fenced to prevent grazing. Before fencing was established, grazing caused vegetation degradation in this area. Grasslands in this area vary in terms of their degradation status due to different grazing intensities (Wang and Ripley, 1997; Jiang et al., 2010). From 2006 to 2019, some degraded sites were gradually recovered. However, the local grassland has a strong soil heterogeneity, which leads to an uneven distribution of soil nutrients within the fencing area (Yang et al., 2021). Likewise, soil salinization also inhibits degraded grassland restoration (Gao et al., 2019). These reasons lead to a heterogeneous restoration of different degraded sites (Gao et al., 2019; Liu et al., 2021). In our study, these plots could be identified by the guideline for grassland degradation assessment (Su and Zhang, 2004; Pellant et al., 2005). We referenced this guideline and previous studies and named these plots as severe degradation status (SD), moderate degradation status (MD), light degradation status (LD), and non-degradation status (ND), respectively (Zeng et al., 2018; Liu et al., 2021; Yang et al., 2021). In detail, *L. chinensis* is the primary dominant species in the ND. With the grassland degradation, the amounts of *L. chinensis* have gradually decreased in the LD. Subsequently, some annual forbs such as *Kochia sieversiana* appears in the MD. Finally, the annual forbs and *Chloris virgata* are the main plant species in the SD (Zeng et al., 2018).

### Field vegetation survey and soil collection

In August 2019, we randomly selected the four 10 × 10 m plots in each degradation status (SD, MD, LD, and ND) within the fencing area. To ensure that representative soils were collected, these plots had at least 200 m intervals, and a 1 × 1 m quadrat was randomly selected in each plot. Therefore, each degradation status has four quadrats. According to previous studies, these sampling sites are reasonable to fully reflect the study area’s characteristics (Pereira et al., 2014; Bowatte et al., 2018). In each quadrat, we harvested all of the plants. The height, density, and coverage of each plant species were measured and recorded in the field. The measurement of these factors is referenced from previous studies in the Tongyu station (Gao et al., 2019; Liu et al., 2021). The plant biomass was measured by clipping each plant species, then drying at 60°C for 48 h to weigh the dried plants.

Subsequently, we collected the experimental soil at the vegetation survey site. After removing the litter of topsoil, 0.5 kg of soil was collected from 0 to 15 cm of topsoil using a soil auger (4 cm diameter). Each quadrat was randomly sampled four times and mixed together as a soil sample for each plot (soil *in situ*). These soils were transported to the laboratory, and the pretreatment and measurement of these soils were the same as the soil with *L. chinensis* (see the section of Soil sampling and measurements). Additionally, 10 kg of soil was taken from each vegetation survey site using the same method to cultivate *L. chinensis*.

We calculated the grassland degradation index (GDI) to confirm the grassland degradation level of each site using the following equation:


$$\begin{array}{l} GDI=\frac{1}{3}\left(\frac{1}{3}P_{1}-\frac{1}{3}P_{2}-\frac{1}{3}P_{3}\right)+\frac{1}{3}\left(\frac{1}{3}STC+\frac{1}{3}STN+\frac{1}{3}STP\right)\\ \;+\frac{1}{3}\left(\frac{1}{2}SW-\frac{1}{2}pH\right) \end{array}$$

Where P_1_, P_2_, and P_3_ are the relative abundances of climax species, annual species, and the salinization indicator species of grassland, respectively; STC, STN, and STP represent the relative concentration of soil total C, N, and phosphorus (P), respectively; SW and pH are the relative soil gravimetric water content and soil pH, respectively. The relative number of this equation was calculated by the actual value of each factor dividing the sum value of this factor across the four degradation statuses. This equation was referenced from previous studies (Zeng et al., 2018; Dong et al., 2020). Likewise, the GDI has been proven to determine the different degradation status of the meadow steppe in the Songnen Plain (Yang et al., 2021). We calculated the GDI of ND, which was 0.14 (Supplementary Table S1). We treat GDI = 0.14 as 100% of grassland (non-degraded grassland), GDI in the range of <60%, 60–80%, and 80–90% represent severe, moderate, and light degradation status, respectively. These thresholds are referenced from a previous study in the Songnen Plain, which is located near Tongyu station conducted in the Songnen Plain (Yang et al., 2021), and our field survey results verified the accuracy of these thresholds. In this area, the field investigation sites were SD (GDI = −0.14, −103% of ND), MD (GDI = 0.10, 72% of ND), and LD (GDI = 0.13, 90% of ND) (Supplementary Table S1).

According to our field vegetation survey results, the plant assemblage types corresponding to the varying degradation statuses were named *Chloris virgata* + *Kochia sieversiana* assemblage, *L. chinensis* + forbs assemblage, *L. chinensis* + *Lespedeza daurica* assemblage, and *L. chinensis* assemblage. Plant density and coverage were gradually increased from SD to ND. In detail, the *C. virgata* + *K. sieversiana* assemblage of SD had low plant diversity and plant biomass. The vegetation type mainly consisted of saline-alkali tolerant plants. The assemblage type of MD is *L. chinensis* + forbs, which has a high plant diversity. In the LD, the main vegetation types were *L. chinensis* and *L. daurica*, and a few *Calamagrostis macrolepis*. *L. chinensis* assemblage of ND had a few plant species, and the dominant *L. chinensis* had the highest biomass (Supplementary Table S1). Moreover, grassland degradation increased soil pH but decreased soil total C, N, and P (Supplementary Table S2).

### Experimental design

In August 2018, 1 kg of *L. chinensis* seeds were randomly collected near the field survey area. In April 2019, these seeds were surface-sterilized and sown in the nursery with sterilized soil, therefore growing in the greenhouse at Jilin Songnen Grassland Ecosystem National Observation and Research Station, China (44° 45′N, 123°45′E). In May 2019, we transplanted these 4-week-old seedlings into pots (12 cm diameter, 14 cm height). Each pot was filled with 1.5 kg of soil from the varying degradation statuses, and three *L. chinensis* seedlings were planted. All pots were placed randomly in the greenhouse. These pots were turned once every 2 weeks, and water was added once per week to ensure plant growth. Overall, we had 8 (4 × 2) treatments from the soil *in situ* and soil with *L. chinensis* and four replicates for each treatment.

### Soil sampling and measurements

After 10 weeks, the soil samples in the pots were collected on August 1, 2019. The rhizosphere soil of *L. chinensis* was collected in each pot using the “shaken-off” method (Aira et al., 2010). These rhizosphere soils were passed through a 2 mm sieve and divided into two subsamples. One subsample of the rhizosphere soil was air-dried to analyze the soil properties (i.e., the C, N, and P contents, soil pH, and gravimetric water content). A total organic carbon analyzer (Vario TOC, Elementar, Germany) was used to measure soil total C, a Kjeldahl apparatus (Kjeltec 8,400, FOSS, Denmark) was used to determine soil total N, and an automatic discontinuous chemical analyzer (Smartchem 450, AMS, Italy) was used to measure soil total P. Soil pH was estimated by a water suspension (water/soil = 5:1) and a pH meter (pH S-3C, INESA, China). Soil gravimetric water content was measured by drying the samples at 105°C to a constant weight.

Another subsample of the rhizosphere soil was immediately stored in liquid nitrogen and transferred to the laboratory. Soil EEAs and microbial community determination were performed simultaneously to avoid the reduction in soil enzyme activity caused by freezing and thawing. We measured the activities of α-1,4-glucosidase (αG), β-1,4-glucosidase (βG), and β-1,4-xylosidase (βX) for the soil C cycle; the activities of L-leucine aminopeptidase (LAP) and β-1,4-N-acetyl-glucosaminidase (NAG) for the soil N cycle; the activity of acid phosphatase (AP) for the soil P cycle. These EEAs were determined by microplate fluorimetric assay (German et al., 2011). The measurement details of extracellular enzyme activities can be found in previous studies (DeForest, 2009; Shi et al., 2018). Soil bacterial and fungal communities were measured using high-throughput sequencing, and the details of soil DNA extraction and high-throughput sequencing are available in the Supplementary material. After quality control, the final total data set retained 3,086 and 1,465 operational taxonomic unit (OTU) numbers and 79,883 and 78,727 average clean reads for bacteria and fungi, respectively.

### Calculation and statistical analyses

To assess the soil microbial characteristics, we tested microbial alpha diversity (Shannon index) between the soil with *L. chinensis* and the soil *in situ* (i.e., without *L. chinensis*). The FAPROTAX was used to predict the functional groups of bacterial communities, and the fungal functional groups were estimated using FUNGuild. Afterward, we selected microbial functional groups closely associated with plant growth and soil nutrient cycling and calculated the relative abundance of these functional groups. The differences in microbial Shannon index and relative abundance were calculated using the one-way ANOVA under the same degradation status. In addition, one-way ANOVA was also used to test the effects of planting *L. chinensis* on the EEAs and soil properties in each degradation status.

To quantify the effect of planting *L. chinensis* on EEAs (i.e., soil *in situ* compared with soil with *L. chinensis*), we used the following formula to calculate the variability index (*VI*) of EEAs:


$$VI=100\%\times \frac{E_{B}-E_{A}}{E_{A}}$$

Where *E_A_* represents the mean EEAs value for soil *in situ*, and *E_B_* represents the average EEAs value for soil with *L. chinensis*. *VI* > 0 or *VI* < 0 represents the percent increase or decrease after planting *L. chinensis*, respectively.

We calculated the Bray-Curtis distance of the six EEAs (αG, βG, βX, LAP, NAG, and AP enzymes) as the soil enzyme composition. Ordinary least squares linear regression was used to evaluate the correlation between microbial Shannon index and soil enzyme composition. We also measured the C cycling enzyme activities (the sum activity of αG, βG, and βX), N cycling enzyme activities (the sum activity of LAP and NAG), and P cycling enzyme activities (AP enzyme activity). Pearson correlation was used to testify to the correlation between microbial relative abundance and soil enzyme activities.

We used the structural equation model (SEM) to analyze the regulation mechanisms of *L. chinensis* on the soil microbial communities and EEAs. The soil with *L. chinensis* and grassland degradation were treated as exogenous variables. Endogenous variables included the composition of bacterial and fungal communities, extracellular enzyme activities, and soil properties. According to the Akaike information criterion (AIC), the core predictors of this study were selected (Burnham and Anderson, 2002). We then used the following factors to evaluate the fit of our models: The goodness of fit of the SEM was evaluated using a root mean squared error of approximation (rmsea<0.05) and a chi-square test (0 < Chi-sq < 2, *p* > 0.05). The comparative fit index (0.97 < cfi < 1) and standardized root mean square residual (srmr<0.05) were used for the best-fitting of each factor (Schermelleh-Engel et al., 2003). Significant differences in all statistical analyses were measured using a *T* test (at an alpha level of 0.05). All statistical analyses were conducted using R 4.0.2 (R Core Team, 2015).

## Results

### *Leymus chinensis* culture affected soil microbial communities and EEAs

**Figure 1:**
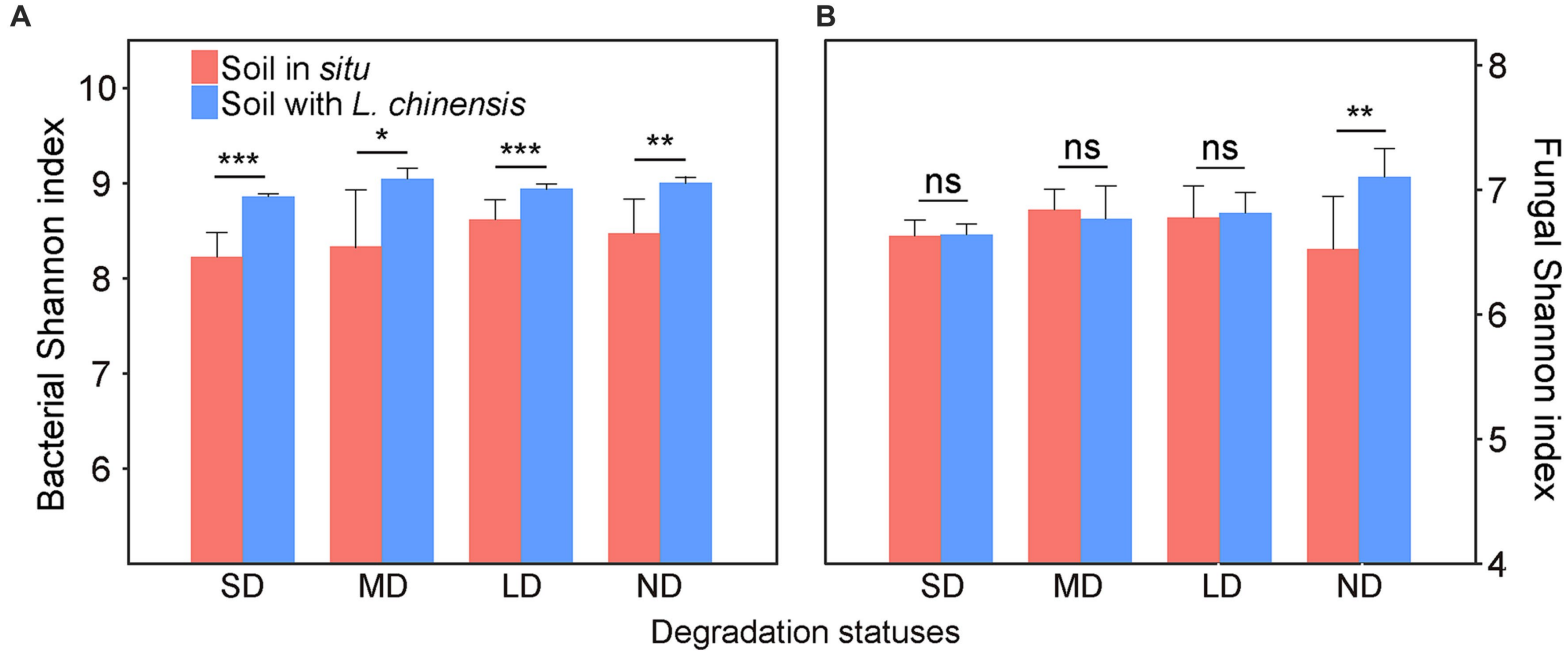
The differences in Shannon index of bacteria **(A)** and fungi **(B)** between the soil *in situ* and soil with *Leymus chinensis* in each degradation status. Vertical bars represent mean ± SE, *n* = 4. SD, severe degradation status; MD, moderate degradation status; LD, light degradation status; ND, non-degradation status. ^***^p < 0.001; ^**^p < 0.01; ^*^p < 0.05; ns, no significant difference.

In the varying degradation statuses, the bacterial Shannon index in the soil with *L. chinensis* was significantly higher than that *in situ* soil (Figure 1A). *L. chinensis* culture significantly increased the fungal Shannon index in the non-degradation status (ND) (Figure 1B).

**Figure 2:**
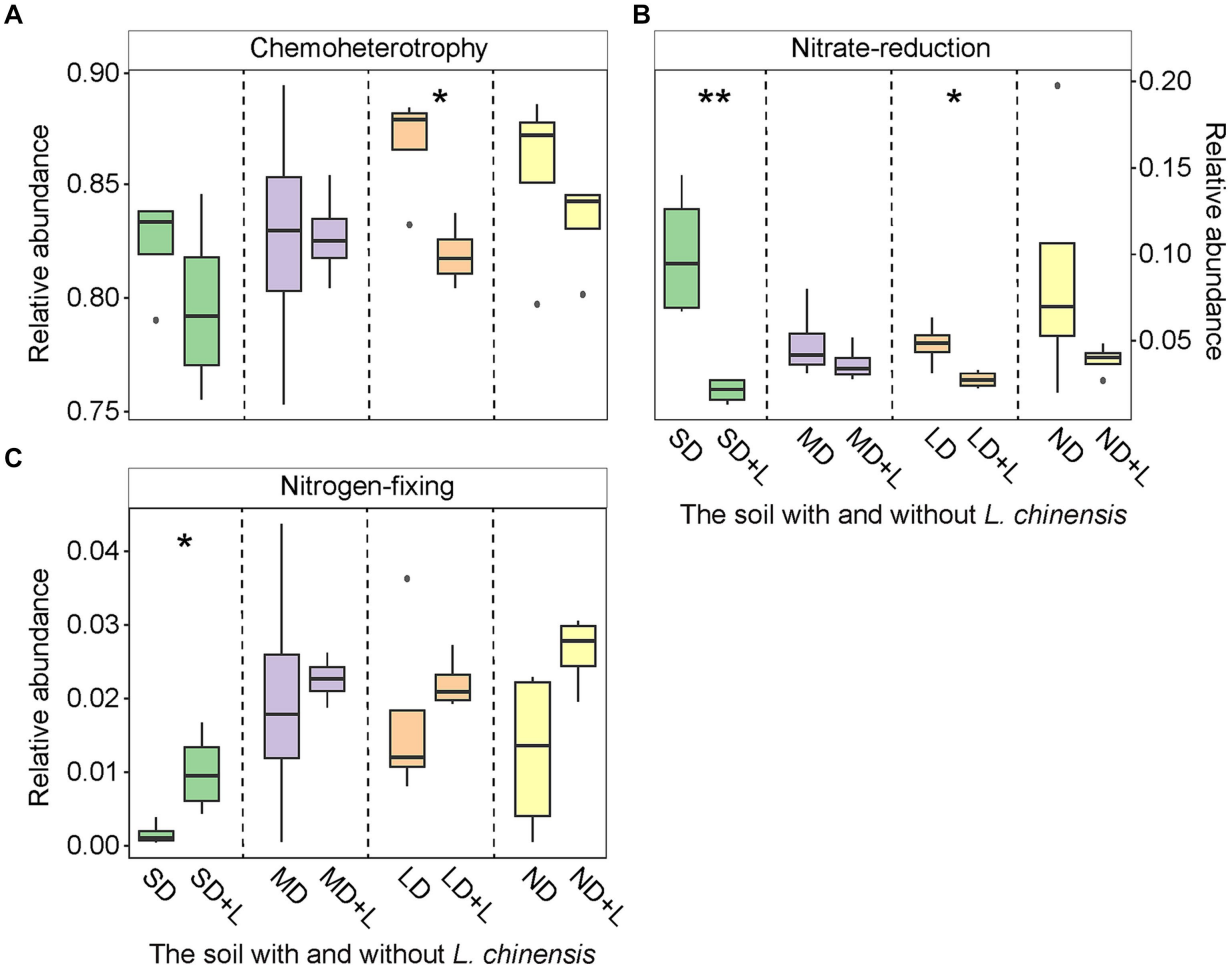
The relative abundance of bacterial Chemoheterotrophy **(A)**, Nitrate-reduction **(B)**, and Nitrogen-fixing **(C)** functional group in the different degradation statuses. The identification of these bacterial functional group is based on the FAPROTAX. Vertical bars represent mean ± SE, n = 4. SD, severe degradation status; MD, moderate degradation status; LD, light degradation status; ND, non-degradation status; SD + L, MD + L, LD + L, and ND + L indicate the corresponding stage after *L. chinensis* plantation. ***p < 0.001; **p < 0.01; *p < 0.05.

**Figure 3:**
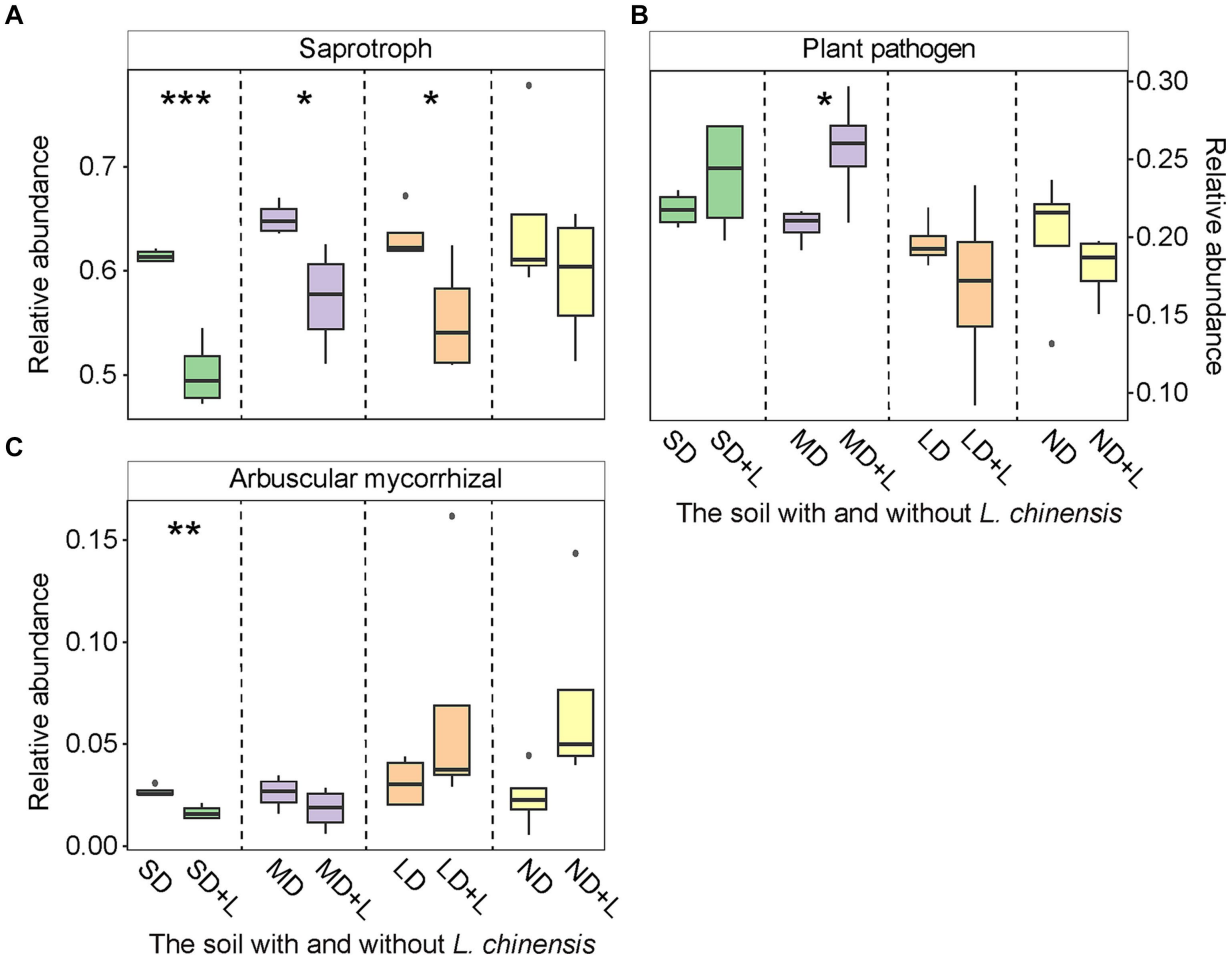
The relative abundance of fungal Saprotroph **(A)**, Plant pathogen **(B)**, and Arbuscular mycorrhizal **(C)** functional group in the different degradation statuses. The identification of these fungal functional group is based on the FUNGuild (Fungi Functional Guild). Vertical bars represent mean ± SE, *n* = 4. SD, severe degradation status; MD, moderate degradation status; LD, light degradation status; ND, non-degradation status; SD + L, MD + L, LD + L, and ND + L indicate the corresponding stage after *L. chinensis* plantation. ***p < 0.001; **p < 0.01; *p < 0.05.

*L. chinensis* culture significantly decreased chemoheterotrophy functional group in the light degradation status (LD) and nitrate-reduction functional group in the severe degradation status (SD) and LD (Figures 2A,B), meanwhile significantly increased nitrogen-fixing functional group in the SD (Figure 2C). Furthermore, saprotroph functional group was significantly decreased in the SD, moderate degradation status (MD), and LD after planting *L. chinensis* (Figure 3A). Besides, *L. chinensis* culture significantly increased the plant pathogen functional group in the MD and significantly decreased the arbuscular mycorrhizal functional group in the SD (Figures 3B,C).

**Figure 4:**
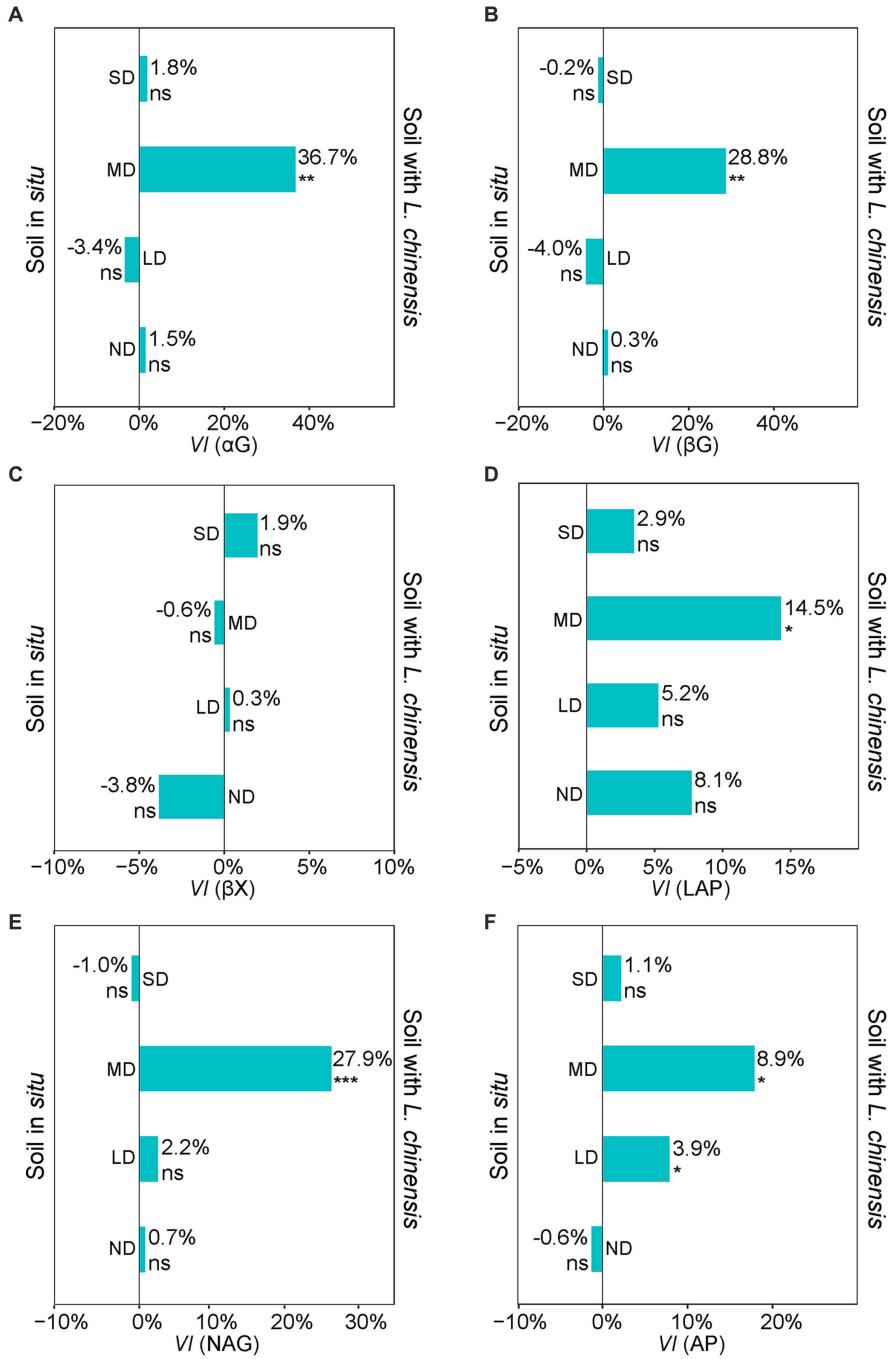
Variability index (*VI*) of αG **(A)**, βG **(B)**, βX **(C)**, LAP **(D)**, NAG **(E)**, and AP **(F)** extracellular enzymes between soil *in situ* and Soil with *L. chinensis*. The percent indicate the mean changes of enzyme activities after we planting *L. chinensis*, *n* = 4. SD, severe degradation status; MD, moderate degradation status; LD, light degradation status; ND, non-degradation status. αG, α-1,4-glucosidase; βG, β-1,4-glucosidase; βX, β-1,4-xylosidase; LAP, L-leucine aminopeptidase; NAG, β-1,4-N-acetylglucosami-nidase; AP, acid phosphatase. ^***^p < 0.001; ^**^p < 0.01; ^*^p < 0.05; ns, no significant difference.

The variability index (*VI*) values showed that *L. chinensis* culture significantly increased the activities of αG, βG, LAP, NAG, and AP in the MD (Figure 4). The αG, βG, LAP, NAG, and AP activities after *L. chinensis* culture were increasing 36.7, 28.8, 14.5, 27.9, and 8.9%, respectively (Figure 4). *L. chinensis* culture also significantly increased the AP activity in the LD (Figure 4F).

### *Leymus chinensis* culture changed the relations between soil microbes and EEAs

**Figure 5:**
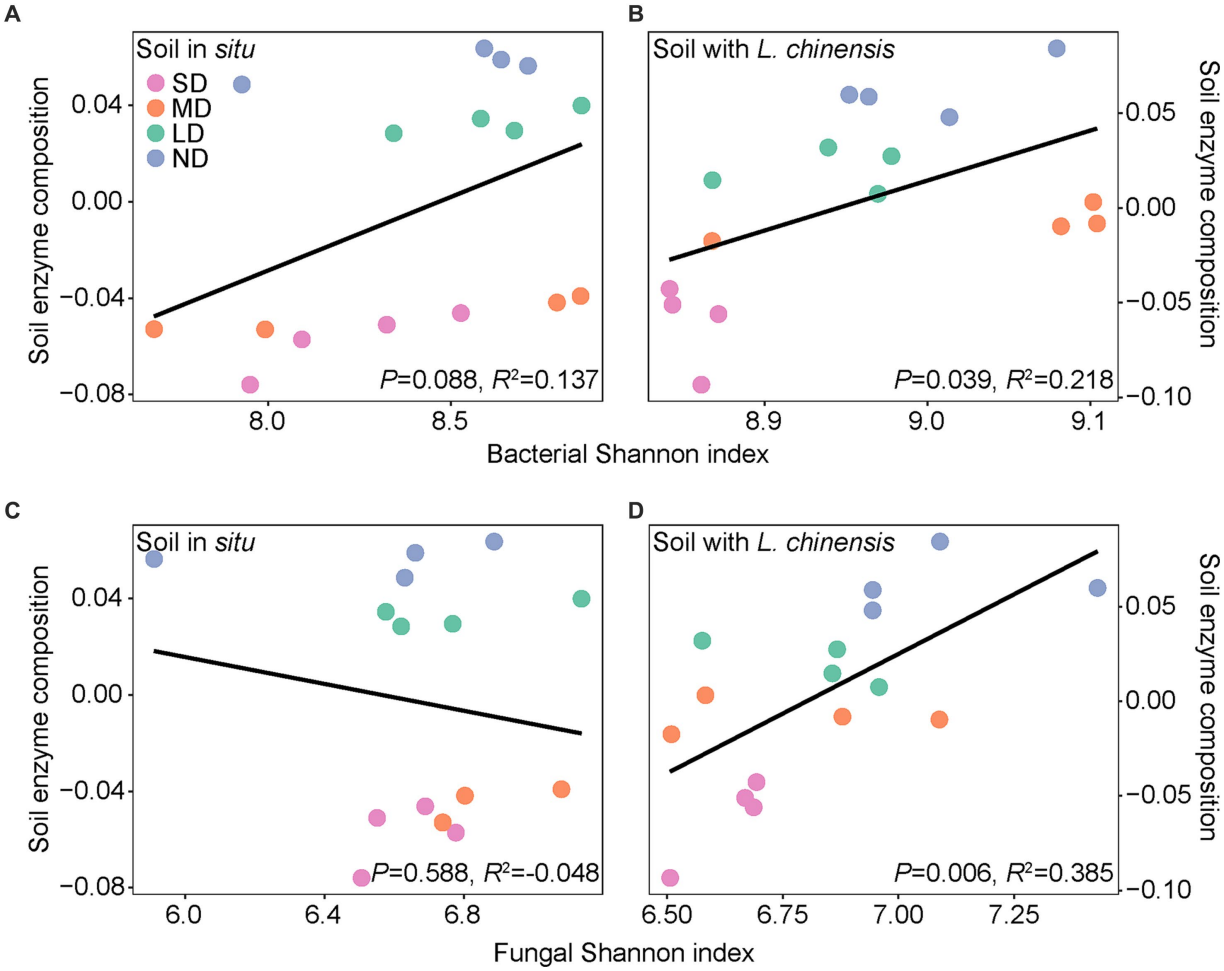
Bacterial **(A,B)** and fungal **(C,D)** Shannon index in relation to soil enzyme composition in the soil *in situ* and soil with *L. chinensis*. The black lines represent the fitted ordinary least squares (OLS) linear regressions. SD, severe degradation status; MD, moderate degradation status; LD, light degradation status; ND, non-degradation status.

**Figure 6:**
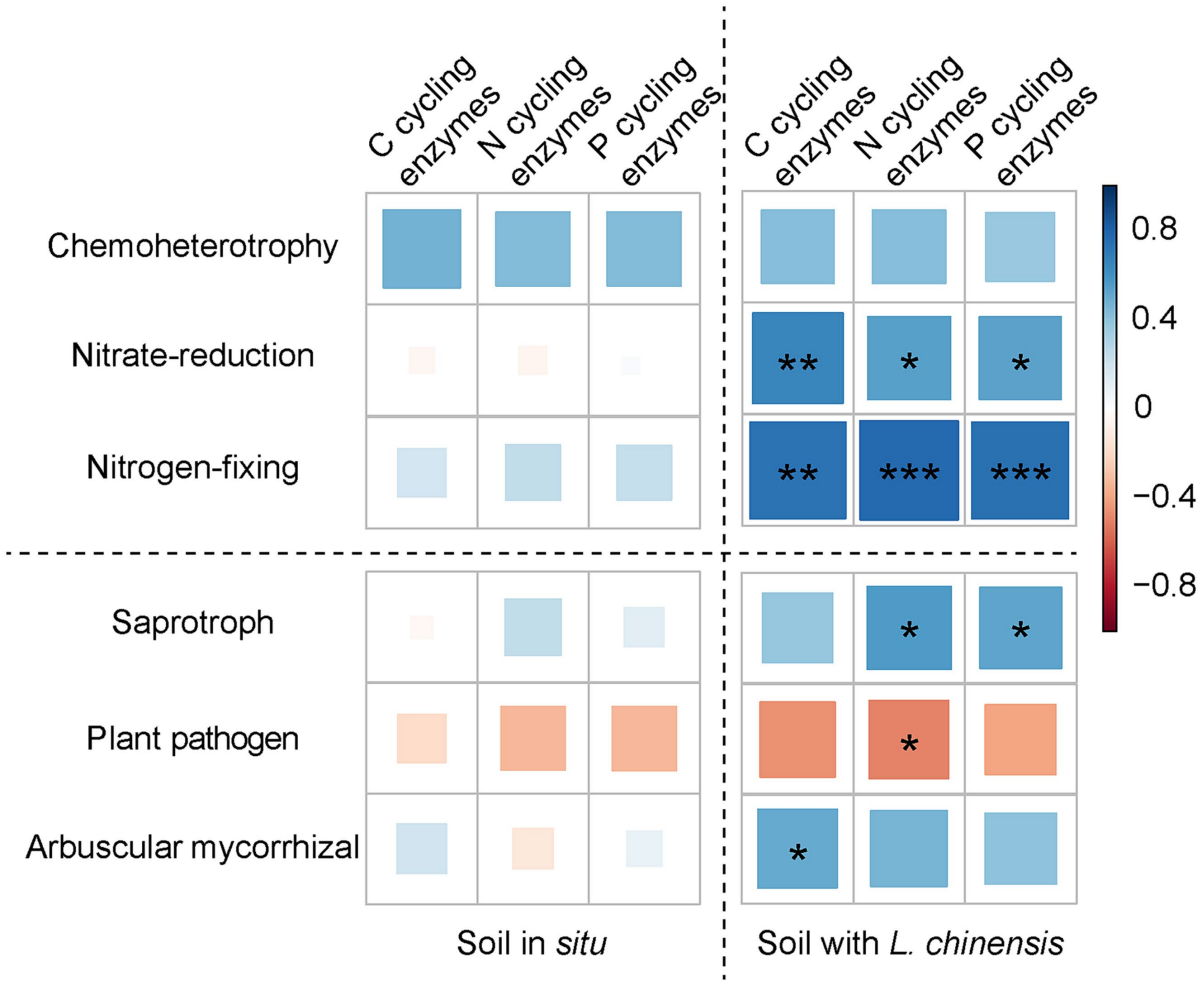
Pearson correlation between soil enzyme activities and microbial functional group in the soil *in situ* and soil with *L. chinensis*. C cycling enzymes, the sum of αG, βG, and βX enzyme activities; N cycling enzymes, the sum of LAP and NAG enzyme activities; P cycling enzymes, AP enzyme activity. ^***^p < 0.001; ^**^p < 0.01; ^*^p < 0.05.

Ordinary least squares linear regression showed that the microbial Shannon index positively correlates with soil enzyme composition after *L. chinensis* culture (Figure 5). Pearson correlation showed that the microbial functional group and soil enzyme activities had no significant correlations in the soil *in situ*. After *L. chinensis* culture, nitrate-reduction functional group and nitrogen-fixing functional group positively correlated with soil enzyme activities. *L. chinensis* culture also enhanced the correlation between fungal functional groups and soil enzyme activities (Figure 6).

### The relationships among the soil microbes, EEAs, and soil properties

**Figure 7:**
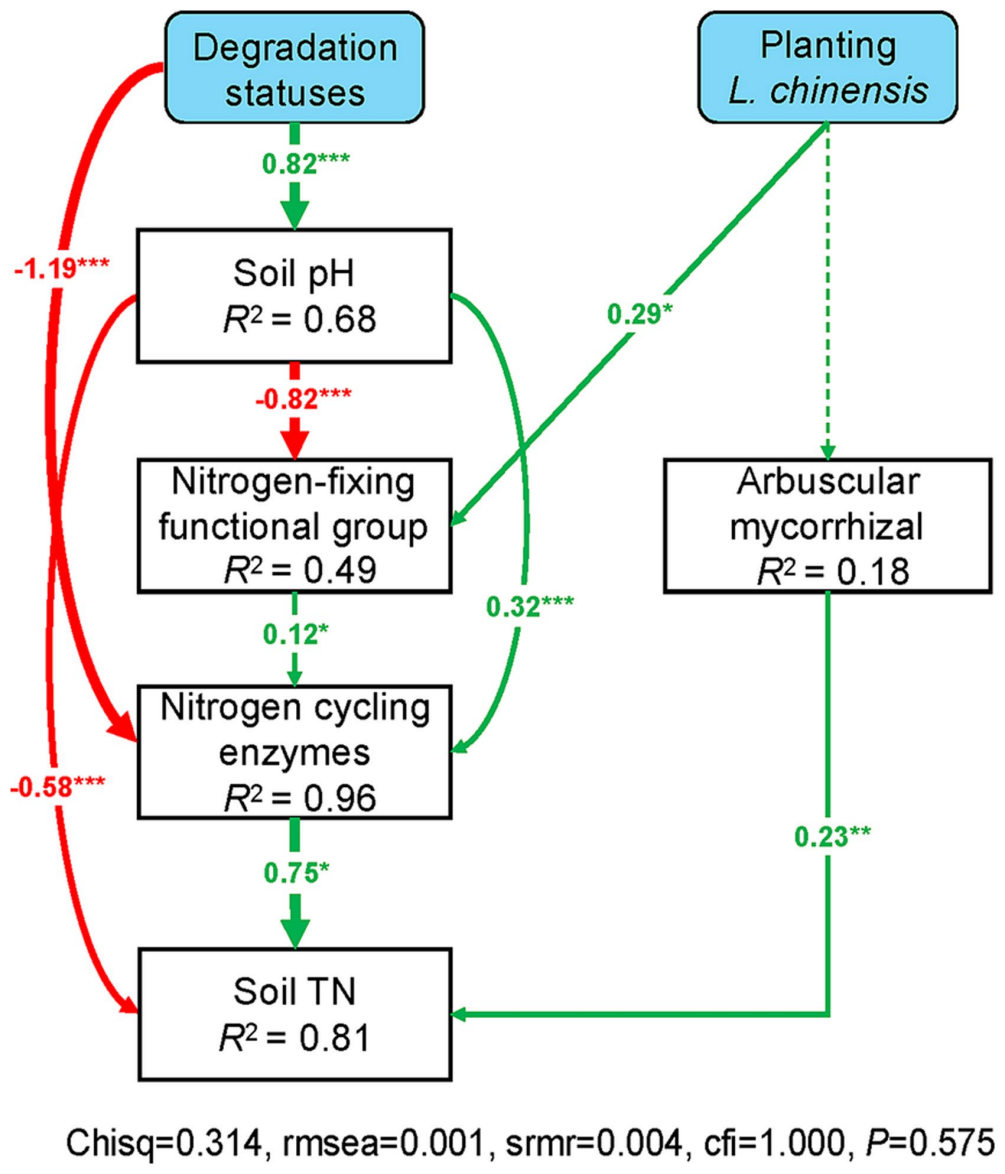
Structural equation models revealed the effects of planting *L. chinensis* on the interaction between soil microbial communities and extracellular enzymes in the degraded grassland. Green and red arrows indicate positive and negative relationships, respectively. Continuous and dashed arrows indicate significant (*p* < 0.05) relationships and no significant (*p* > 0.05) relationships, respectively. Numbers on arrows are standardized path coefficients. The thickness of the arrow indicates the strength of the relationship. *R*^2^ values associated with response variables indicate the proportion of variation explained by relationships with other variables. Nitrogen-fixing and Arbuscular mycorrhizal indicate the relative abundance of these microbial functional group. Nitrogen cycling enzymes indicate the sum of LAP and NAG enzyme activities. Soil TN indicate soil total nitrogen. ^***^p < 0.001; ^**^p < 0.01; ^*^p < 0.05.

The SEM results showed that grassland degradation negatively affects the nitrogen-fixing functional group and N cycling enzyme activities via increasing soil pH. Grassland degradation also directly decreased N cycling enzyme activities, and the increasing soil pH directly decreased soil total N. Likewise, the nitrogen-fixing functional group had a positive effect on soil total N by regulating N cycling enzyme activities, and arbuscular mycorrhizal functional group directly affected soil total N (Figure 7). Moreover, *L. chinensis* culture positively affected the nitrogen-fixing functional group, thus positively regulating N cycling enzyme activities and soil total N in the degraded grassland (Figure 7).

## Discussion

### *Leymus chinensis* culture influenced soil microbes and EEAs

Our results showed that *L. chinensis* culture promoted bacterial alpha diversity in the varying degradation statuses (Figure 1A). These results were similar to a previous study indicating that reseeding forage affected bacterial communities in the degraded grassland (Wang et al., 2017). Our results may be due to the higher soil pH and lower soil N and P concentrations caused by grassland degradation (Supplementary Table S2). On the one hand, the high soil pH can decline the activities of fungal communities but increase bacterial community activities (Rousk et al., 2010), thus enhancing the bacterial community in response to plant growth. On the other hand, the lower N and P content in the degraded grassland may promote plants regulating microorganisms to obtain soil nutrients, and the high soil pH accelerates the bacteria-mediated rapid nutrition turnover in soil (Vitousek et al., 2010; de Vries et al., 2012). Furthermore, *L. chinensis* is the pioneer plant and dominant species in the Songnen grassland (Gao et al., 2019; Liu et al., 2021), and the growth of *L. chinensis* may modify the severe soil environment of degraded grasslands (Wang and Ripley, 1997; Jing et al., 2015; Zhang et al., 2022). Accordingly, the growth of *L. chinensis* may alleviate the negative influence of severe soil environment (such as soil salinization) on soil microbes, which may be the reason for the increasing bacterial alpha diversity after planting *L. chinensis*. Additionally, *L. chinensis* culture enhanced fungal alpha diversity in the non-degradation status (Figure 1B). Generally, fungal communities were more active in the late successional stage of grassland (Kardol et al., 2006). In the climax community, dominant species critically interact with fungal communities (Kardol et al., 2007). For example, the symbiosis between dominant species and mycorrhizal fungi promotes plant community stability (Kardol et al., 2006). The increasing biomass of dominant species can result in soil fungal pathogen accumulation (Reynolds et al., 2003). In our study, the non-degradation status is a climax community constructed with *L. chinensis*. Therefore, the growth of *L. chinensis* in the non-degradation status soil may potentially increase the fungal alpha diversity.

We found a decreasing trend of chemoheterotrophy and nitrate-reduction functional group after planting *L. chinensis*. Likewise, the abundance of nitrogen-fixing functional group trends to be increased after planting *L. chinensis* (Figure 2). In detail, the chemoheterotrophy functional group is widely distributed in the soil, and these bacterial taxa are mainly involved in the degradation process of organic matter (Wang et al., 2023). In our study, the soil litter was removed before the *L. chinensis* culture. This method reduced the substrate for soil microbes, which may be the reason for the decreasing abundance of chemoheterotrophy functional group. Likewise, the removal of soil litter also caused a decrease in the abundance of saprotroph functional group. The nitrate-reduction usually occurs in an anaerobic environment (Silver et al., 2001). In the field, soil compaction caused by grassland degradation creates the conditions for microbial anaerobic metabolism (Akiyama and Kawamura, 2007). However, the field soil was transferred to the pots, which may have disrupted the anaerobic environment of the soil. Therefore, the abundance of nitrate-reduction functional group may be lower in the pots. The nitrogen-fixing functional group can be classified into symbiotic and asymbiotic bacteria (Kennedy and Islam, 2001). Generally, symbiotic nitrogen-fixing bacteria are mainly associated with legume forage (Udvardi and Poole, 2013). Nevertheless, as the pioneer plants of grassland restoration, gramineous plants can increase asymbiotic bacteria activities to obtain soil N nutrients (Minamisawa et al., 2004; Sun et al., 2022). Finally, *L. chinensis* culture may increase the abundance of nitrogen-fixing functional group.

Our findings showed that *L. chinensis* culture enhanced soil extracellular enzyme activities (EEAs) only in the moderate degradation status (Figure 4). These results support the view that the varying degradation statuses may lead to the different responses of soil EEAs to plant growth (Burns et al., 2013; Liu et al., 2019). In detail, the severe soil environment (e.g., the higher soil salinization) in the severe degradation status may not be suitable for the growth of *L. chinensis* (Jiang et al., 2010), thus potentially limiting the regulation effects of *L. chinensis* on belowground functions (Kardol and Wardle, 2010). Meanwhile, the high soil pH in the severe degradation status may lead to the soil EEAs losing their optimal reaction conditions (Frankenberger and Johanson, 1982). This effect directly inhibited the activities of soil EEAs (Zuccarini et al., 2023), therefore inhibiting the increasing soil EEAs caused by planting *L. chinensis*. Subsequently, moderate degraded grassland has higher soil nutrients and lower soil pH than severe degraded grassland (Jiang et al., 2010; Fry et al., 2017). This soil environment in the moderate degradation status creates conditions for the invasion and growth of *L. chinensis* (Wang and Ripley, 1997; Zhang et al., 2022). Finally, planting *L. chinensis* may increase EEAs through root exudates (Zhang et al., 2015; Steinauer et al., 2016; Liu et al., 2019). Moreover, in the light and non-degradation status, *L. chinensis* is the dominant species and has the highest biomass among the plant communities (Zhang et al., 2022). This plant assemblage composition resulted in *L. chinensis* specifically regulating the soil environment and EEAs for a long time (Liu et al., 2021). Accordingly, we emphasize that *L. chinensis* culture in the moderate degradation status has high recovery efficacy on soil EEAs. The recovery effect of forage culture on soil functions may depend on a suitable degradation status.

### *Leymus chinensis* culture enhanced the interaction between soil microbes and EEAs

Our results showed that planting *L. chinensis* enhanced the positive correlation between microbial diversity and soil extracellular enzyme composition (Figure 5). In detail, extracellular enzymes are produced by microorganisms, which drive the degradation of soil organic matter into inorganic nutrients (Wallenstein and Burns, 2011). However, due to the high nutrient costs for the production of extracellular enzymes, the severe soil environment in degraded grassland (e.g., soil nutrients limitation and high salinization) usually limits the enzyme production of soil microbes (Allison et al., 2010; Burns et al., 2013). Subsequently, this severe soil environment increases the belowground biomass of plants to absorb soil nutrients (Mimmo et al., 2018). As a result, the higher root biomass produces more root exudates, thus providing more resources (such as C sources) to regulate microbial communities, finally enhancing the correlation between soil microbes and EEAs (Brzostek et al., 2014; Schnecker et al., 2015; Mimmo et al., 2018).

Moreover, we found that planting *L. chinensis* increased the positive correlation between soil EEAs and nitrate-reduction and nitrogen-fixing functional group (Figure 6), which indicated that *L. chinensis* could promote the soil N cycle functions of degraded grassland soil. Firstly, nitrate-reduction and nitrogen-fixing functional groups are highly correlated to soil N cycle, and these microbial taxa regulate soil N cycle via EEAs (Kennedy and Islam, 2001; Silver et al., 2001; Liu et al., 2021). Secondly, the degraded grassland soil usually has low nutrient concentration, especially the soil N content (Hawkes et al., 2005; Gong et al., 2021). To ensure the successful colonization and growth of plants, plants in degraded grassland had a strong regulation capacity on soil microbes for more soil nutrients (Tharayil et al., 2013; Ye et al., 2021). Lastly, as the pioneer species of degraded grassland, *L. chinensis* may promote the activity of microbial communities via increasing labile C inputs to the soil (Parraga-Aguado et al., 2013; Pii et al., 2015), thus potentially enhancing the interaction between microbial communities and soil EEAs.

### *Leymus chinensis* culture enhanced soil N content via regulating soil microbies and EEAs

The SEM results demonstrated that *L. chinensis* culture promoted soil total N by enhancing the relationships between nitrogen-fixing functional group and N cycling enzymes (Figure 6). Our finding was similar to previous studies that plant growth positively affects soil microorganisms and EEAs in the grassland (Wang et al., 2017; Chuan et al., 2020; Liu et al., 2021). Meanwhile, the direct effects of EEAs on soil total N may be due to the low concentration of soil N in the degraded grassland (Marklein and Houlton, 2011; Lü et al., 2013). Accordingly, we conclude that planting *L. chinensis* may promote soil N content by enhancing the interaction between nitrogen-fixing functional group and soil EEAs in the degraded grassland. Moreover, the high soil pH caused by grassland degradation interfered with the regulating effects of *L. chinensis* (Figure 6). In detail, the high soil pH can inhibit the activities of nitrogen-fixing microbes, thus negatively impacting the activity of N cycling enzymes (Fan et al., 2018; Liu and Zhang, 2019; Luo et al., 2020). Likewise, the severe soil environment, such as soil salinization and compaction, which inhibits the soil EEAs (Singh K, 2015; Singh JS, 2015; Teng et al., 2020). Finally, we suggest that the high soil pH may alleviate the positive effects of *L. chinensis* on soil microbes and EEAs in the degraded grassland. Notably, our study only performed 10 weeks of pot experiment. In the field area, the recovery of forage culture on soil functions may be more complex due to environmental disturbances (such as drought or increased temperature). Forage culture may need to consider the soil condition of degraded grassland to ensure the effectiveness of forage cultivation in recovering soil functions.

## Conclusion

Our study discussed how *L. chinensis* culture regulated soil microbial communities and extracellular enzymes in degraded grassland soils. We found that planting *L. chinensis* promoted the microbial-mediated soil EEAs in the moderate degradation status, indicating that the restoration effects of the grass culture on soil EEAs depended on the degradation status in the grassland. Subsequently, *L. chinensis* promoted the interaction between the soil nitrogen-fixing functional group and soil extracellular enzymes, thus enhancing the N content in the degraded grassland soil. Therefore, the grass culture benefited the recovery of soil functions, especially soil N cycling. Overall, this study provided evidence for improving soil microbial community structure and restoring soil function. It emphasized the selection of the plant species for restoration of grasslands according to the restoration effects of microbial functional groups and soil function.

## Data availability statement

The datasets presented in this study can be found in online repositories. The names of the repository/repositories and accession number(s) can be found below: https://www.ncbi.nlm.nih.gov/, PRJNA1012694.

## Author contributions

MZ: Conceptualization, Data curation, Formal analysis, Writing – original draft, Writing – review & editing. ZL: Investigation, Software, Supervision, Validation, Writing – review & editing. BZ: Investigation, Supervision, Validation, Writing – review & editing. RZ: Investigation, Supervision, Validation, Writing – review & editing. FX: Conceptualization, Funding acquisition, Methodology, Writing – original draft, Writing – review & editing.
