# Supplementary material for: Planting grass enhances relations between soil microbes and enzyme activities and restores soil functions in a degraded grassland
Source: Front Microbiol. 2024 Feb 15;15:1290849. doi: 10.3389/fmicb.2024.1290849 (PMC10903263; doi:10.3389/fmicb.2024.1290849)
Supplement: Supplementary file 1 [file Table_1.DOCX]

**Table S1** The vegetation characteristics of the varying degradation statuses in the Songnen grassland (mean±*SE*, n=4).

| Degradation statuses | Plant assemblage type | Main plant species | Above ground  biomass (g · m ^-2^) | Height (cm) | Density  (# · m ^-2^) | Coverage (%) |
| --- | --- | --- | --- | --- | --- | --- |
|  |  |  |  |  |  |  |
| Severe degradation status  GDI=-0.14 (-103%) | *Chloris virgata* +  *Kochia sieversiana* | *C. virgata* | 18.72 ± 11.67 | 17.38 ± 4.79 | 192.50 ± 140.44 | 34.50 ± 24.31 |
|  |  | *K. sieversiana* | 82.61 ± 12.09 | 22.93 ± 7.01 | 528.00 ± 338.83 | 63.75 ± 22.87 |
|  |  | *Artemisia scoparia* | 15.21 ± 7.55 | 26.03 ± 6.82 | 72.50 ± 37.38 | 15.00 ± 8.29 |
|  |  |  |  |  |  |  |
| Moderate degradation status  GDI=0.10 (72%) | *Leymus chinensis* +  forbs | *L. chinensis* | 148.69 ± 15.41 | 34.63 ± 9.17 | 285.00 ± 46.35 | 55.00 ± 14.72 |
|  |  | *Calamagrostis macrolepis* | 3.45 ± 2.78 | 33.53 ± 9.98 | 4.25 ± 2.30 | 8.00 ± 5.12 |
|  |  | *Lespedeza daurica* | 7.99 ± 6.08 | 20.32 ± 7.85 | 10.50 ± 8.70 | 3.75 ± 1.99 |
|  |  | *K. sieversiana* | 2.07 ± 1.01 | 27.83 ± 9.75 | 1.50 ± 0.91 | 1.38 ± 0.43 |
|  |  | *A. scoparia* | 1.31 ± 0.45 | 25.61 ± 9.29 | 4.50 ± 2.65 | 1.50 ± 1.00 |
|  |  | *Allium mongolicum* | 1.33 ± 0.53 | 30.00 ± 5.89 | 2.50 ± 1.65 | 1.38 ± 0.80 |
|  |  |  |  |  |  |  |
| Light degradation status  GDI=0.13 (90%) | *L. chinensis* +  *L. daurica* | *L. chinensis* | 227.22 ± 36.47 | 49.45 ± 9.45 | 384.00 ± 36.37 | 48.75 ± 14.45 |
|  |  | *C. macrolepis* | 33.94 ± 41.58 | 56.40 ± 8.54 | 62.00 ± 75.15 | 8.75 ± 14.36 |
|  |  | *L. daurica* | 70.27 ± 20.43 | 48.90 ± 12.81 | 76.50 ± 38.99 | 31.25 ± 16.52 |
|  |  |  |  |  |  |  |
| Non-degradation status  GDI=0.14 (100%) | *L. chinensis* | *L. chinensis* | 234.64 ± 35.61 | 46.60 ± 8.82 | 431.00 ± 164.90 | 85.00 ± 8.16 |
|  |  | *Carex duriuscula* | 24.89 ± 12.96 | 17.20 ± 5.41 | 612.00 ± 272.82 | 30.00 ± 7.07 |

Note: GDI, grassland degradation index.

**Table S2** One-way ANOVA of the effects of planting *L. chinensis* on soil properties in the varying degradation statuses (mean±*SE*, n=4)

| Treatments | | TC (mg·kg ^-1^) | TN (mg·kg ^-1^) | TP (mg·kg ^-1^) | pH | SW (%) |
| --- | --- | --- | --- | --- | --- | --- |
| Severe degradation status | Soil in *situ* | 9032.50±381.96 a | 155.95±6.62 a | 202.80±6.57 a | 9.71±0.11 a | 10.04±0.43 a |
|  | Soil with *L. chinensis* | 9067.50±409.01 a | 154.75±11.25 a | 201.00±7.70 a | 9.62±0.08 a | 5.20±0.74 b |
|  |  |  |  |  |  |  |
| Moderate degradation status | Soil in *situ* | 9475.00±190.00 a | 167.70±1.94 a | 201.75±6.58 a | 8.77±0.43 a | 8.86±0.74 a |
|  | Soil with *L. chinensis* | 9627.50±188.75 a | 168.60±6.02 a | 211.25±12.91 a | 8.57±0.18 a | 4.75±1.35 b |
|  |  |  |  |  |  |  |
| Light degradation status | Soil in *situ* | 9280.00±385.57 a | 177.50±4.36 a | 225.80±4.43 a | 8.13±0.29 a | 8.58±1.15 a |
|  | Soil with *L. chinensis* | 9750.00±118.04 a | 185.65±5.13 a | 226.35±6.40 a | 8.26±0.04 a | 6.78±0.96 a |
|  |  |  |  |  |  |  |
| Non-degradation status | Soil in *situ* | 9630.00±392.60 a | 183.40±7.91 a | 230.30±3.40 a | 8.46±0.13 a | 11.37±0.67 a |
|  | Soil with *L. chinensis* | 9415.00±332.72 a | 185.28±6.02 a | 228.65±7.35 a | 8.32±0.13 a | 8.36±1.90 b |

Note: TC, soil total carbon; TN, soil total nitrogen; TP, soil total phosphorus; pH, soil pH; SW, soil water content. Different letters indicate significant difference (*P* < 0.05) between soil in *situ* and soil with *L. chinensis*.

**Table S3** The composition of soil extracellular enzymes (Bray-Curtis distance) between soil in *situ* and soil with *L. chinensis*.

| Degradation statuses | Bray-Curtis distance | |
| --- | --- | --- |
|  | Soil in *situ* | Soil with *L. chinensis* |
| Severe degradation status | -0.0759 | -0.0511 |
|  | -0.0510 | -0.0560 |
|  | -0.0462 | -0.0933 |
|  | -0.0571 | -0.0427 |
| Moderate degradation status | -0.0529 | 0.0031 |
|  | -0.0391 | -0.0174 |
|  | -0.0418 | -0.0082 |
|  | -0.0530 | -0.0097 |
| Light degradation status | 0.0399 | 0.0274 |
|  | 0.0284 | 0.0147 |
|  | 0.0295 | 0.0075 |
|  | 0.0345 | 0.0319 |
| Non-degradation status | 0.0487 | 0.0480 |
|  | 0.0564 | 0.0588 |
|  | 0.0636 | 0.0843 |
|  | 0.0590 | 0.0598 |

**Supplementary material for high-throughput sequencing and analyses**

The soil DNA was extracted from 0.25 g of each replicate soil sample using the MoBio Powersoil TM DNA Isolation Kits (MoBio Laboratories, Inc., USA) according to the manufacturer’s instructions. The extracted DNA was evaluated using 1% agarose gel, and DNA concentrations were measured using a NanoDrop 2000 to confirm the final DNA concentration of each sample. The F: 5′-ACTCCTACGGGAGGCAGCA-3′, R: 5′-GGACTACHVGGGTWTCTAAT-3′ was used to amplify the V3-V4 regions of the bacterial rRNA. The F: 5′-CTTGGTCATTTAGAGGAAGTAA-3′, R: 5′-GCTGCGTTCTTCATCGATGC -3′ was used to amplify the ITS1 primer pairs, regions of the fungal rRNA. To distinguish the target gene amplicons originating from the different samples, a unique 8-bp barcode was added to the 5′ end of both the forward and reverse primers. Amplicon paired-end sequencing was implemented using the Illumina MiSeq platform at Genesky Biotechnologies Inc. (Shanghai, China).

Sequencing data was analyzed according to acknowledged guidelines (Schöler et al., 2017; Vestergaard et al., 2017). Initially, raw data were merged with the lowest overlap of 10 bp using FLASH (Magoc and Salzberg 2011), and then the low-quality sequences (quality score < 20) or sequences shorter than 100 bp were discarded using Trim Galore. The primer sequences, barcodes, unresolved nucleotides, and adapter were removed from the sequences by using Mothur (Schloss et al., 2009), and the sequences with more than two mismatches to primers were eliminated from the sample. Chimeras were subsequently checked and removed using USEARCH (Edgar 2010). The remaining high-quality sequences were converted to FASTA format and concatenated into a single file. Finally, high-quality reads were clustered at a cut-off of 97% identity using the UPARSE pipeline (Edgar 2013), and the clusters with singleton sequences were removed. The taxonomies of unique operational taxonomic units (OTUs) were annotated using the RDP Classifier (Wang et al., 2007). To reduce the impact of sequencing depth variation among the different samples, each sample was then rarefied to the same sequence depth for downstream analyses.

The raw bacterial and fungal reads were deposited into the National Center for Biotechnology Information (NCBI) Sequence Read Archive (SRA) database under accession number PRJNA1012694.

**References**

Edgar, R. C. (2010). Search and clustering orders of magnitude faster than BLAST. Bioinformatics 26(19), 2460-2461. https://doi.org/10.1093/bioinformatics/btq461

Edgar, R. C. (2013). UPARSE: highly accurate OTU sequences from microbial amplicon reads. Nat Methods 10(10), 996-998. https://doi.org/10.1038/nmeth.2604

Magoc, T., Salzberg, S. L. (2011). FLASH: fast length adjustment of short reads to improve genome assemblies. Bioinformatics 27(21), 2957-2963. https://doi.org/10.1093/bioinformatics/btr507

Schloss, P. D., Westcott, S. L., Ryabin, T., Hall, J. R., Hartmann, M., Hollister, E. B., Lesniewski, R. A., Oakley, B. B., Parks, D. H., Robinson, C. J. (2009). Introducing mothur: open-source, platform-independent, community-supported software for describing and comparing microbial communities. Appl. Environ. Microbiol. 75(23), 7537-7541. https://doi.org/10.1128/AEM.01541-09

Schöler, A., Jacquiod, S., Vestergaard, G., Schulz, S., Schloter, M. (2017). Analysis of soil microbial communities based on amplicon sequencing of marker genes. Bio. Fert. Soils 53, 485-489. https://doi.org/10.1007/s00374-017-1205-1

Vestergaard, G., Schulz, S., Schöler, A., Schloter, M. (2017). Making big data smart-how to use metagenomics to understand soil quality. Bio. Fert. Soils 53, 479-484. https://doi.org/10.1007/s00374-017-1191-3

Wang, Q., Garrity, G. M., Tiedje, J. M., Cole, J. R. (2007). Naive Bayesian classifier for rapid assignment of rRNA sequences into the new bacterial taxonomy. Appl. Environ. Microbiol. 73, 5261-5267. https://doi.org/10.1128/AEM.00062-07
